# 911. Psychosocial Needs of the Burn Survivor: What We Know, Don't Know and Should Know

**DOI:** 10.1093/jbcr/irag033.436

**Published:** 2026-04-06

**Authors:** Ashley E Honea, Brianna Blackwell, Sandra J Cramolini, Jill L Sproul, Karen J Richey, Kevin N Foster

**Affiliations:** Diane & Bruce Halle Arizona Burn Center - Valleywise Health, Phoenix, AZ; Phoenix Society for Burn Survivors, Markle, IN; Phoenix Society for Burn Survivors, Knoxville, TN; Phoenix Society for Burn Survivors, San Jose, CA; Diane & Bruce Halle Arizona Burn Center - Valleywise Health, Phoenix, AZ; Diane & Bruce Halle Arizona Burn Center - Valleywise Health, Phoenix, AZ

## Abstract

**Introduction:**

Burn survivors face significant changes in function, appearance, and social settings that can lead to body image dissatisfaction, stigmatization, and isolation, resulting in high risk for long-term psychosocial complications. While research shows that quality of life and psychosocial adjustment correlate with available support levels, many burn center employees report feeling uncomfortable addressing patients' psychosocial needs. This educational project aims to provide survivor-informed, evidence-based education about burn survivors' specific psychosocial needs to increase burn team awareness and understanding. In doing this, we will enable staff to appropriately identify, discuss, respond to, and better support survivors while evaluating the education's effectiveness in improving staff understanding and confidence in providing psychosocial care.

**Methods:**

Staff completed a mandatory 32-question pre-training assessment measuring confidence (5-point Likert scale) and knowledge (multiple-choice) in delivering survivor-informed psychosocial education to burn survivors and caregivers. Demographics included gender, age, position, work area, tenure, and number of psychoeducational presentations completed. After a one-hour evidence-based presentation on burn survivor psychosocial needs, staff completed the same post-training assessment.

**Results:**

There was a total of 176 participants that took the pre-test and 110 post-tests. The average level of confidence across all 8 domains was 2.72 pretest vs 3.54 posttest, representing a 33% increase. The greatest gains were seen in understanding components of trauma (75%↑), discussing sexual intimacy (44% ↑) and talking about body image (28% ↑). The mean pre-test knowledge score was 80% vs 86% post-test. Individuals showed an improvement across 20 of the 21 domains.

**Conclusions:**

This survivor-informed education significantly improved staff confidence and knowledge in addressing burn survivors' psychosocial needs. While limited to one center, the data demonstrated meaningful improvement. Enhanced understanding of long-term psychosocial complications enables burn teams to better support survivors through their recovery journey, improving care delivery models.

**Applicability of Research to Practice:**

These results support the provision of survivor-informed, evidence-based education on burn survivors’ specific psychosocial needs to burn team members. A better understanding of long-term psychosocial complications is essential to enhance burn care delivery models.

**Funding for the study:**

N/A.

